# Intermittent hypoxia promotes melanoma lung metastasis via oxidative stress and inflammation responses in a mouse model of obstructive sleep apnea

**DOI:** 10.1186/s12931-018-0727-x

**Published:** 2018-02-12

**Authors:** Lian Li, Fangyuan Ren, Chao Qi, Leiqian Xu, Yinshan Fang, Maoli Liang, Jing Feng, Baoyuan Chen, Wen Ning, Jie Cao

**Affiliations:** 1 Respiratory Department, Tianjin Medical University General Hospital, Tianjin, China; 2 State Key Laboratory of Medicinal Chemical Biology, College of Life Sciences, Nankai University, Tianjin, China

**Keywords:** Obstructive sleep apnea, Intermittent hypoxia, Lung metastasis, Oxidative stress, Inflammation, Antioxidant tempol

## Abstract

**Background:**

Recently, increased tumor incidence and cancer-related mortality have been reported among patients with obstructive sleep apnea (OSA). Intermittent hypoxia (IH), the hallmark feature of OSA, contributes to the metastasis of tumors. However, the molecular mechanisms by which tumor metastasis is accelerated by OSA-like IH remain to be elucidated.

**Methods:**

C57BL/6 J male mice were subjected to intravenous injection of B16F10 melanoma cells before receiving IH treatment. Then, the animals were randomly distributed into three groups (*n* = 8 each): normoxia (N) group, IH group, and antioxidant tempol group (IHT, exposed to IH after treatment with tempol). After the mice were sacrificed, the number and weight of lung metastatic colonies were assessed. The lung tissues with tumor metastasis were analyzed for markers of oxidative stress and inflammation and for HIF-1α using western blotting and real-time PCR (qRT-PCR). The level of reactive oxygen species (ROS) in B16F10 cell was also assessed after N, IH and IH with tempol treatments.

**Results:**

Compared with normoxia, IH significantly increased the number and weight of mouse lung metastatic colonies. Treatment of B16F10 cells with IH significantly enhanced ROS generation. Lung tissues with tumor metastasis provided evidence of increased oxidative stress, as assessed by p22^phox^ and SOD mRNA levels and the NRF2 protein level, as well as increased inflammation, as assessed by TNF-α and IL-6 mRNA levels and the NF-κB P65 protein level. HIF-1α protein levels were increased in response to IH treatment. Tempol, an important antioxidant, ameliorated IH-induced melanoma lung metastasis in mice and reduced oxidative stress and inflammation responses.

**Conclusions:**

These results support the hypothesis that oxidative stress and inflammation responses play an important role in the pathogenesis of OSA-like IH-induced melanoma lung metastasis in mice. Antioxidant intervention provides a novel strategy for the prevention and treatment of cancer in OSA populations.

## Background

Obstructive sleep apnea (OSA), a common sleep breathing disorder, is characterized by recurrent upper airway obstruction triggered by complete or partial upper airway collapse during sleep. It can lead to intermittent hypoxia (IH), sleep fragmentation and daytime sleepiness. OSA affects at least 4–10% of adults and has been reported as a critical predisposing factor for cardiovascular morbidity [1–4], metabolic disorder [5, 6], hypertension and cognitive dysfunction [7, 8]. Currently, several epidemiological studies have demonstrated that cancer progression and cancer-related mortality are accelerated in patients with OSA [9–14]. Therefore, IH is considered to be a major characteristic factor of OSA for promoting tumor invasion and metastasis [15, 16]. Animal studies also indicate that OSA-like IH enhances the growth, invasion and metastasis of tumors [17–20]. However, the underlying mechanisms for this OSA-like IH-induced tumor metastasis are not completely understood.

Hypoxia is increasingly recognized as an important feature of the intra-tumoral microenvironment, and it can induce the growth and metastasis of tumors via the up-regulation of hypoxia-inducible factor-1α (HIF-1α) [21]. However, in contrast to continuous hypoxia, IH in patients with OSA is a unique physiological state with a phase of post-hypoxic re-oxygenation (ROX). It is characterized by higher frequency, more serious hypoxia and larger variation in blood oxygen saturation. The relationship between OSA-like IH and tumors has been explored in recent years. The hypothesis that OSA-like IH results in the development and progression of tumors has been proposed in several investigations [17, 22–25]. OSA-like IH includes a phase of post-hypoxic ROX, which results in the production of reactive oxygen species (ROS) and increases oxidative stress and inflammation responses. These represent important pathological links between OSA and injuries of multiple organs including the myocardium [26, 27], carotid body [28, 29], adrenal gland [30] and brain [31, 32]. Recently, Gutsche et al. showed that IH increases pro-metastatic gene expression by activating NF-κB in inflammatory breast cancer cells [25]. Here, we hypothesized that oxidative stress and inflammation responses may play important roles in a mouse model of OSA-like IH-induced tumor metastasis.

4-Hydroxy-2,2,6,6-tetramethylpiperidine-N-oxyl (tempol), a well-known antioxidant, promotes the clearance of ROS and protects mitochondria from oxidative damage. It has been reported that tempol can reduce the atherosclerosis associated with metabolic syndrome by reducing vascular inflammation and repressing NADPH-2 oxidase expression [33]. It also exerts neuroprotective effects by reducing superoxide anions and preventing peroxynitrite-associated inflammation [34–36]. Recently, tempol has been confirmed to attenuate apoptotic signals in endothelial cells from IH-exposed rats by decreasing oxidative stress and inflammation injury [37]. Therefore, it is reasonable to hypothesize that tempol may attenuate IH-induced tumor metastasis by decreasing oxidative stress and inflammation responses. This study was designed to investigate the enhancement of OSA-like IH-induced melanoma lung metastasis by oxidative stress and inflammation responses, and to assess the interventional role of the antioxidant tempol.

## Methods

### Animals

C57BL/6 J male mice with the age of 8 weeks were purchased from Model Animal Research Center of Nanjing University. All mice were housed and cared for in standard cages and provided access to food and water freely at the General Hospital of Tianjin Medical University. The animals, after being injected with melanoma cells, were randomly distributed into three groups (*n* = 8): N group, IH group and IHT group, which was treated with tempol (Sigma-Aldrich, St. Louis, MO, USA). All animal experimental protocols were approved by the Animal Care and Use Committee at General Hospital of Tianjin Medical University and were performed in accordance with the guidelines outlined by the committee.

### Melanoma cell culture

Murine melanoma B16F10 cells were obtained from the Laboratory of Lung Development and Diseases at Nankai University (Tianjin, China) with the American Type Culture Collection (ATCC; Manassas, VA, USA) as the original source. These cells were cultured in DMEM supplemented with 10% fetal bovine serum (Gibco, Carlsbad, CA, USA) and antibiotics in a 37 °C incubator with a humidified atmosphere containing 5% CO_2_.

### Establishment of the induced melanoma lung metastasis model

Briefly, the mice were anaesthetized with chloral hydrate (Sangon Biotech, Shanghai, China) and then intravenously injected with 1 × 10^5^ B16F10 cells diluted in 100 μL of PBS via tail vein. The number and weight of metastatic colonies per lung were assessed after 3 weeks.

### Animal exposure to IH and tempol administration

The animal model of IH was established according to a previously described protocol [38]. The mice were exposed to IH for 6 h/day during the light period (10 AM–4 PM) for 21 consecutive days in a specialized plexiglas chamber (23 cm × 20 cm × 12 cm = 5520 cm^3^ ≈ 5.5 L) with 8 mice per cage. The flow of nitrogen (99.99% N_2_, hypoxia phase) or compressed air (air, ROX phase) was modulated by a gas control delivery system in the chamber to maintain a nadir oxygen concentration of 5% and an IH cycle. The alternating cycle lasted for 2 min, and consisted of hypoxia and ROX phases. Gas flow and O_2_ concentration were monitored continuously with an O_2_ analyzer (CY-12C, Meicheng, China). During the hypoxia phase, the O_2_ concentration in the chamber was decreased to 5% within 20 s by infusion of N_2_ and remained at that concentration for 30 s. During the ROX phase, the O_2_ concentration was rapidly increased to 21% within 10 s by flushing the chamber with compressed clean air, which was then sustained for 60 s. In general, 180 cycles were completed during the exposure period (10 AM–4 PM) every day. Mice exposed to the N condition were used as the control group. Mice in the IHT group received an intraperitoneal injection of 1 ml of 10% (*w/v*) tempol per kilogram body weight prior to IH exposure every day, throughout the whole exposure period of 3 weeks.

### Metastasis assessment

Five lung lobes were carefully separated from the mice in the IH-induced melanoma metastasis model. The number of metastatic colonies per lung was counted after anterior and posterior image acquisition. Metastatic colonies were then separated from the lung lobes, and the weight of the metastases per lung was measured.

### RNA isolation and qRT-PCR

Samples of mouse lung tumor tissue from the three groups (*n* = 8) were homogenized in Trizol. RNA isolation and qRT-PCR analysis were performed as previously described [39]. The expression of p22^phox^, SOD, TNF-α and IL-6 was determined using a SYBR Green Master Mix kit (Roche Diagnostics, Indianapolis, IN, USA). Mouse β-actin was used as an internal control. Gene expression was quantified relative to the mRNA levels from mouse lung tumor tissue, which was amplified in parallel. The sequences of specific primer pairs are listed in Table 1.

**Table 1:** Real-Time PCR primers used in this study

Genes	Forward primer 5′-3′	Reverse primer 5′-3′
*p22* ^*phox*^	AAGTACCTGACCGCTGTGG	AGGTAGATCACACTGGCAATG
*SOD*	TTATGATGGGCACTGCAAAGAA	ACTGCCTTTAGAGAACCAGCC
*TNF-α*	CCAAACCAGCCTGACAACTT	TCTAGCATGCTCCACCACTG
*IL-6*	GTGTAGCACAACTTCCAATTACGAA	GGAATTTCCGCCTCGAGTCT
*β-actin*	AGGCCAACCGTGAAAAGATG	AGAGCATAGCCCTCGTAGATGG

### Western blot analysis

Proteins were extracted from mouse lung tumor tissue according to standard protocols as described previously [40]. Protein concentrations were measured using a BCA assay kit (Pierce, Rockford, IL, USA). The proteins were separated by SDS-PAGE and electroblotted onto PVDF membranes (Millipore, Bedford, MA, USA). These membranes were blocked with 5% skim milk for 1 h at room temperature and then incubated with primary antibodies at 4 °C overnight. The primary antibodies, including HIF-1α (1:200; Novus Biologicals, Littleton, CO, USA), NF-E2-related factor 2 (NRF2) (1:200; ProteinTech Group, Chicago, IL, USA), NF-κB P65 (1:5000; Santa Cruz Biotechnology Inc., Santa Cruz, CA, USA) and β-actin (1:5000; Santa Cruz Biotechnology Inc., Santa Cruz, CA, USA), were used to probe the target proteins. Goat anti-mouse IgG-HRP or goat anti-rabbit IgG-HRP (1:5000; Santa Cruz Biotechnology Inc., Santa Cruz, CA, USA) were used as secondary antibodies and were incubated with the membranes for 2 h at room temperature. Bands were visualized with ECL reagents. The intensity of protein bands on the scanned images was analyzed with a Tanon Gis digital image analytical system. The accumulation of HIF-1α, NRF2 and NF-κB P65 was quantified as the ratio of the band intensities for the target protein and β-actin.

### ROS detection in vitro

B16F10 cells were seeded in 60 mm culture dishes and incubated for 12 h with DMEM supplemented with 10% fetal bovine serum. After two washes with PBS, the culture medium was replaced with 2 ml DMEM supplemented with 2% fetal bovine serum. The cells were treated with N, IH (30 s 5% O_2_–90 s 21% O_2_) and IH with tempol for 8 h/d, for 2 d. ROS generation was determined using a Reactive Oxygen Species Assay Kit (Beyotime, China). The cells were washed twice with pre-warmed PBS and then incubated with DCFH-DA (10 mM) at 37 °C for 30 min. After two washes with PBS, the cells were collected with 1 ml lysis buffer. Then, the clear supernatant was transferred to a 96-well plate after centrifugation at 12000 rpm for 10 min. Relative fluorescence was read with a BioTek Cytation 5 System (BioTek, Winooski, VT) set at 488 nm excitation and 525 nm emission. All assays were repeated in triplicate.

### Statistical analysis

SPSS version 16.0 (SPSS Inc., Chicago, IL) software package was used for statistical analysis. All data are presented as Mean ± SEM. Comparisons between several groups were analyzed using one-way ANOVA. Difference between experimental and control group was analyzed using Student’s t test. The statistically significant difference was considered at *P* < 0.05.

## Results

### OSA-like IH promotes melanoma lung metastasis

**Fig. 1:**
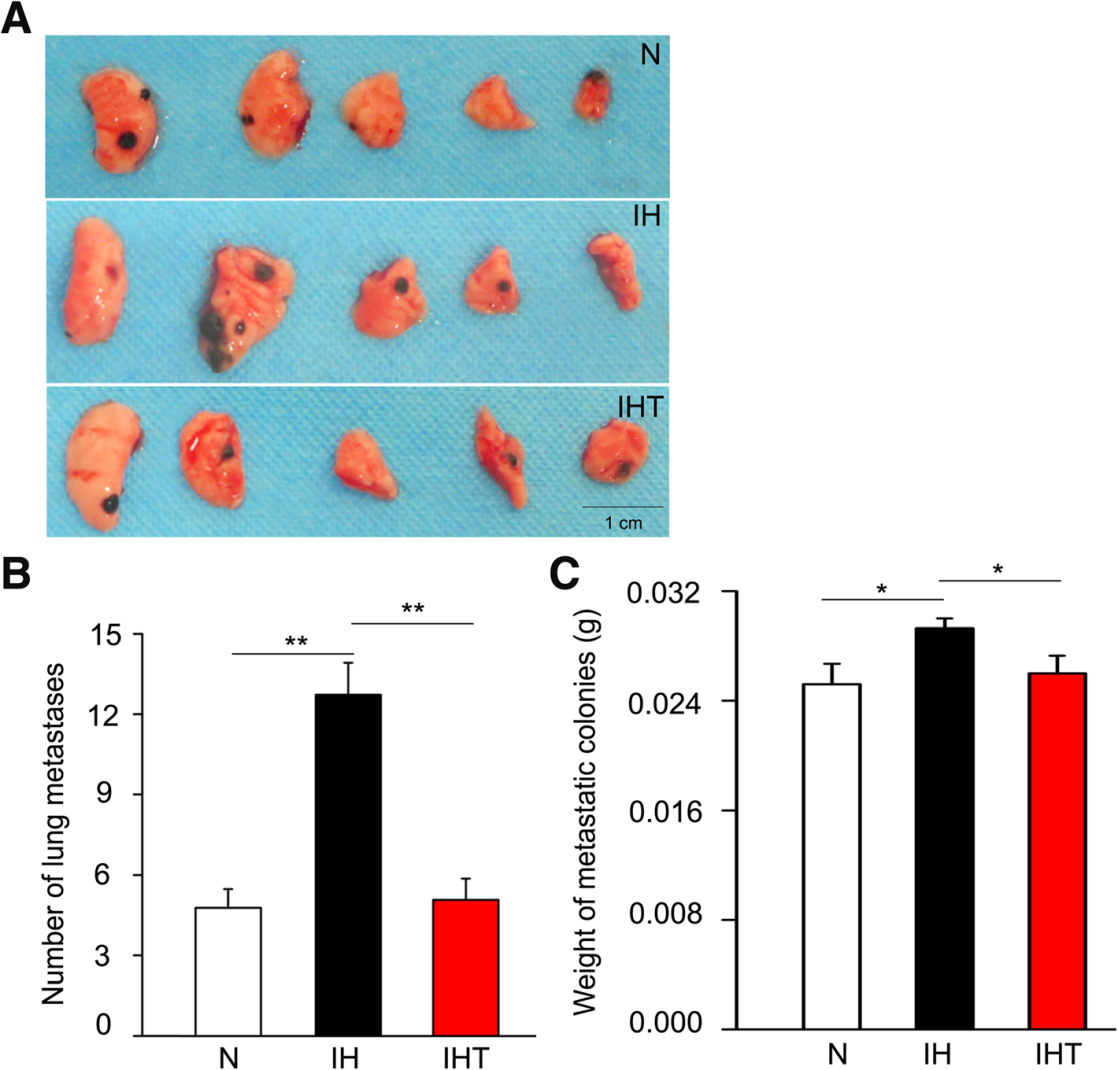
Effects of OSA-like IH on melanoma lung metastasis. **a** Example of lung metastasis, visible as black dots on the surface of 5 lung lobes from mice subjected to N, IH, or IHT treatment. **b** Total number of melanoma lung metastatic colonies observed in the N, IH, or IHT groups. **P < 0.01. **c** The weight of melanoma lung metastatic colonies was assessed. *P < 0.05. *n* = 8 per group. All data are expressed as the mean ± SEM

The number of lung metastatic melanomas was determined at day 21 after the exposure to N, IH and IHT conditions. The number of metastatic melanomas per lung in the IH group (12.8 ± 1.5) was significantly higher than that in the N group (4.8 ± 0.7) (Fig. 1a, b) (*P* < 0.01). However, the number of metastatic melanomas per lung in the IHT group (5.1 ± 0.8) was lower than that in the IH group (Fig. 1a, b) (*P* < 0.01).

In addition, compared to that of the N group, the weight of lung metastases in the IH group was significantly increased (Fig. 1c) (*P* < 0.05). The weight of lung metastases in the IHT group was decreased when compared with that of the IH group (Fig. 1c) (*P* < 0.05). These findings suggested that OSA-like IH increased melanoma metastasis in the lung, and this enhancement effect was prevented by pretreatment with the antioxidant tempol (Fig. 1a, b, c).

### Increased ROS generation in murine melanoma cells after exposure to OSA-like IH

**Fig. 2:**
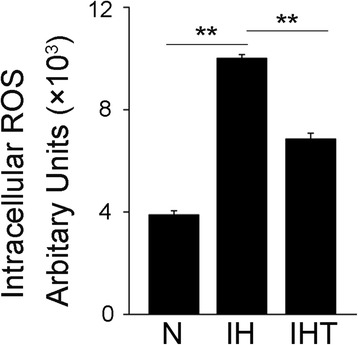
Effect of OSA-like IH on ROS generation in murine melanoma cells. B16F10 cells were treated with N, IH, or IHT, stained with DCFH-DA, and then analyzed with a BioTek Cytation 5 System. **P < 0.01. The experiments were performed three times

OSA-like IH can be interrupted by posthypoxic reoxygenation, which results in the generation of O_2_ free radicals and oxidative stress. In current study, we first investigated the effect of OSA-like IH exposure on ROS levels in murine melanoma B16F10 cells. As shown in Fig. 2, we found that compared with normoxia, OSA-like IH markedly increased ROS levels (Fig. 2) (*P* < 0.05). However, the antioxidant tempol effectively diminishes this OSA-like IH-induced ROS production (Fig. 2).

### Activated oxidative stress response in melanoma lung metastasis mouse model after exposure to OSA-like IH

**Fig. 3:**
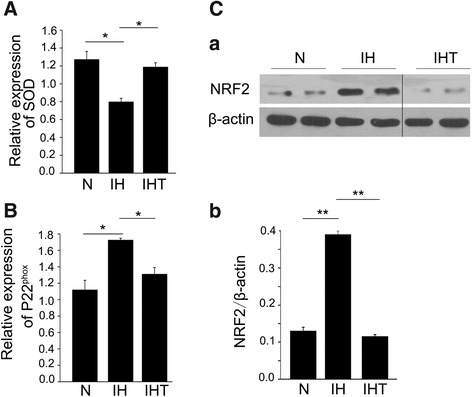
Effect of OSA-like IH on oxidative stress responses in melanoma lung metastasis mice. qRT-PCR analysis of SOD (**A**) and p22^phox^ (**B**) mRNA expression (*n* = 6 per group) and western blotting analysis of NRF2 protein expression (**C** *a*) in tumor tissue from mouse lungs after OSA-like IH exposure and/or tempol treatment (*n* = 3 per group). The expression level of NRF2 was evaluated by densitometric analysis of western blot bands (**C** *b*). All data are expressed as the mean ± SEM. *P < 0.05, **P < 0.01

ROS generation can activate oxidative stress and inflammation responses. Accordingly, we investigated the roles of oxidative stress and inflammation responses in the mouse model of OSA-like IH-induced tumor metastasis. To assess the activation of the oxidative stress response in the IH-induced lung metastasis mouse model, the mRNA expression levels of SOD and p22^phox^ were evaluated by qRT-PCR. The transcription factor NF-E2-related factor 2 (NRF2), which is related to oxidative stress, was evaluated by western blotting. The mRNA expression level of SOD in the IH group was significantly decreased compared with that in the N group (Fig. 3A) (*P* < 0.05). The mRNA expression level of p22^phox^ in the IH group was also significantly increased compared with that in the N group (Fig. 3B) (*P* <  0.05). The protein expression level of NRF2 was significantly increased in the IH group when compared with that in the N group (Fig. 3C a, b) (*P* < 0.01). These results indicated that OSA-like IH enhanced oxidative stress in the melanoma lung metastasis mouse model. As expected, oxidative stress in the IHT group was significantly reduced compared with that in the IH group (Fig. 3A, B, C).

### Activated inflammation response in melanoma lung metastasis mouse model after exposure to OSA-like IH

**Fig. 4:**
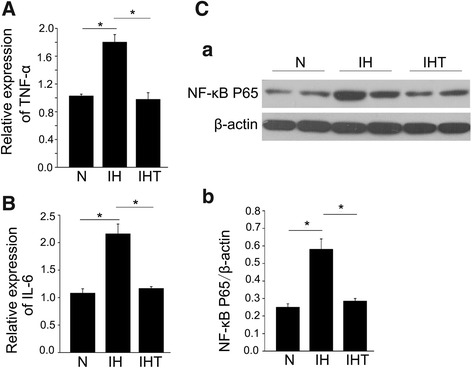
Effect of OSA-like IH on the inflammatory response in melanoma lung metastasis mice. qRT-PCR analysis of TNF-α (**A**) and IL-6 (**B**) mRNA expression (n = 6 per group) and western blotting analysis of NF-κB P65 protein expression (**C** *a*) in tumor tissue from mouse lungs after OSA-like IH exposure and/or tempol treatment (n = 3 per group). The expression level of NF-κB P65 (**C** *b*) was evaluated by densitometric analysis of western blot bands. All data are expressed as the mean ± SEM. *P < 0.05

Next, to examine the activation of the inflammation response in the OSA-like IH-induced lung metastasis mouse model, the mRNA expression levels of TNF-α and IL-6, as well as the protein level of the inflammation response transcription factor NF-κB P65, were evaluated by qRT-PCR and western blotting, respectively. The mRNA expression levels of TNF-α and IL-6 in IH group were significantly increased when compared with those in the N group (Fig. 4A, B) (*P* < 0.05). The protein expression level of NF-κB P65 in the IH group was significantly increased when compared with that in the N group (Fig. 4C a, b) (*P* < 0.05). These results suggested that OSA-like IH activated the inflammation response in this melanoma lung metastasis mouse model. Similarly, inflammation in the IHT group was significantly attenuated when compared with that in the IH group (Fig. 4A, B, C).

### Increased HIF-1α expression in melanoma lung metastasis mouse model after exposure to OSA-like IH

**Fig. 5:**
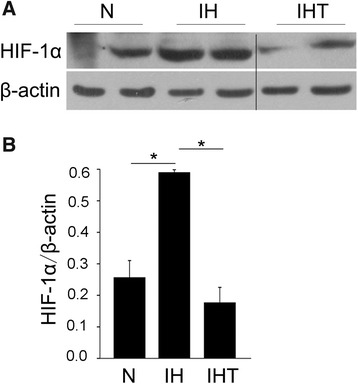
Effect of OSA-like IH on HIF-1α expression in a melanoma lung metastasis mouse model. **a** Western blotting analysis of HIF-1α expression in tumor tissue from mouse lungs after OSA-like IH exposure and/or tempol treatment (n = 3 per group). **b** The expression level of HIF-1α was evaluated by densitometric analysis of western blot bands. All data are expressed as the mean ± SEM. *P < 0.05

HIF-1α is an important transcription factor for responding to hypoxia, and plays an important role in tumor metastasis. The protein expression level of HIF-1α in the IH group was significantly higher than that in the N group (*P* <  0.05). However, the protein expression level of HIF-1α in the IHT group was significantly lower than that in the IH group (Fig. 5a, b) (*P* <  0.05), suggesting that tempol treatment attenuates the OSA-like IH-induced expression of HIF-1α in this melanoma lung metastasis mouse model.

## Discussion

Epidemiological studies have demonstrated that OSA can promote the development and mortality of cancers. Recently, the relationship between OSA-like IH and tumors has only been explored in animals. In the present study, OSA-like IH exposure increased melanoma metastasis in the lung, which is remarkably similar to previous reports [18], consistently with the observation that patients with OSA have a close relationship with high cancer incidence and cancer-related mortality [10–14].

OSA-like IH may be interrupted by ROX. In the ROX phase, excessive ROS, primarily derived from activated NADPH oxidase, can lead to oxidant/antioxidant imbalance, and cause radical-induced oxidation and damage. Many studies have confirmed that oxidative stress is associated with the occurrence and progression of tumors [41]. Herein, we have reported that OSA-like IH enhanced ROS generation in B16F10 cells. The mRNA expression level of the enzymatic antioxidant SOD was significantly lower in the IH group than in the N group. In addition, the mRNA expression level of NADPH oxidase p22^phox^ in the IH group was significantly increased when compared with that in the N group. NRF2, which can be activated by IH, is a key transcription factor for responding to oxidative stress. In the OSA-like IH-induced lung metastasis mouse model, the protein expression level of NRF2 in the IH group was significantly elevated compared with that in the N group. These results suggested that oxidative stress might be the mechanism underlying the melanoma lung metastasis induced by OSA-like IH.

Oxidative stress promotes inflammation responses, and in turn, inflammation enhances oxidative stress. NF-κB is another transcription factor activated by ROS, and it acts as a master regulator of the transcriptional responses to inflammation [42, 43]. Up-regulation of inflammatory molecules or cytokines is associated with tumor proliferation, invasiveness and metastasis [44, 45]. Gutsche et al. showed that in inflammatory breast cancer cells, IH induced the expression of multiple pro-metastatic genes via NF-κB [25]. In our study, we also verified that OSA-like IH significantly increased the protein level of NF-κB in a melanoma lung metastasis mouse model. The mRNA levels of inflammatory markers, including TNF-α and IL-6, were significantly induced by OSA-like IH exposure. Inflammatory responses activated by oxidative stress may be a potential mechanism underlying the melanoma lung metastasis induced by OSA-like IH.

Excessive ROS can be eliminated by exogenous antioxidants in several animal models [46, 47]. Tempol, a redox-cycling nitroxyl radical, can scavenge excessive ROS and increase SOD activity, and is thus considered to be a promising antioxidant for clinical and experimental application. It exerts neuroprotective effects by reducing superoxide anions and attenuating peroxynitrite-associated inflammation. Previous studies have demonstrated that tempol plays a protective role in cardiovascular disease, neurological disorders and cancer by reducing oxidative stress or inflammation. In our OSA-like IH-induced lung metastasis mouse model, tempol repressed the IH-induced oxidative stress and inflammation responses, as well as melanoma lung metastasis. In the future, antioxidant intervention may be an effective strategy for treating OSA-related cancer patients.

HIF-1α is an important transcription factor for the adaptive response to hypoxia. HIF-1α protein accumulates under hypoxic conditions, and is degraded after each reoxygenation [48]. In our study, the protein level of HIF-1α in the IH group was significantly higher than that in the N group, which was consistent with previous studies [24, 49, 50]. IH can induce ROS generation and cause oxidative stress responses. Tempol, known as an antioxidant, promotes the clearance of ROS and protects mitochondria from oxidative damage. Previous reports have shown that tempol or other antioxidants can prevent HIF-1α-mediated cancer progression [37, 51–53]. Consistent with these reports, our findings suggest that antioxidant tempol attenuated melanoma lung metastasis and oxidative stress, as well as decreased HIF-1α expression. Therefore, our study indicates that increased ROS production and oxidative stress induced by OSA-like IH promote melanoma lung metastasis, which is in part regulated through up-regulation of HIF-1α expression. However, other studies have demonstrated that the stabilization of HIF-1α under hypoxia or IH is independent of oxidative stress [25, 54, 55]. These conflicting reports indicate that further study is necessary to confirm the relationship between HIF-1α and oxidative stress.

## Conclusions

These results suggest that the oxidative stress and inflammation responses induced by IH exposure play important roles in the pathogenesis of tumor metastasis, and the use of antioxidants such as tempol may potentially serve as a unique strategy for OSA-related cancer patients.
